# Examining the Treatment Efficacy of PEERS in Japan: Improving Social Skills Among Adolescents with Autism Spectrum Disorder

**DOI:** 10.1007/s10803-019-04325-1

**Published:** 2019-12-10

**Authors:** Tomoko Yamada, Yui Miura, Manabu Oi, Nozomi Akatsuka, Kazumi Tanaka, Naotake Tsukidate, Tomoka Yamamoto, Hiroko Okuno, Mariko Nakanishi, Masako Taniike, Ikuko Mohri, Elizabeth A. Laugeson

**Affiliations:** 1 United Graduate School of Child Development, Osaka University, Kanazawa University, Hamamatsu University School of Medicine, Chiba University and University of Fukui, 2-2 Yamadaoka, Suita, Osaka 565-0871 Japan; 2 United Graduate School of Child Development, Osaka University, Kanazawa University, Hamamatsu University School of Medicine, Chiba University and University of Fukui, 13-1-D Takaramachi, Kanazawa, Ishikawa 920-8640 Japan; 3 Ehime University, 3 Bukyo, Mastuyama, Ehime 790-8577 Japan; 4 Hirakata Board of Education, 1-1-1 Kurumazuka, Hirakata, Osaka 573-1159 Japan; 5 Yamanashi Eiwa College, 888 Yokone, Kofu, Yamanashi 400-8555 Japan; 6 Semel Institute for Neuroscience and Human Behavior, University of California Los Angeles, 760 Westwood Plaza, Ste.48-243B, Los Angeles, CA 90024 USA

**Keywords:** Social skills training, Autism spectrum disorder, PEERS, Adolescents, Friendship

## Abstract

This study examines the efficacy of the Japanese version of the Program for the Education and Enrichment of Relational Skills (PEERS), which focuses on improving social functioning through making friends and maintaining good relationships for adolescents with autism spectrum disorder (ASD) without intellectual disabilities. Originally developed in the United States, PEERS is one of the few evidence-based social skills training programs for youth with ASD. The present study shows that with linguistic and cultural modifications, PEERS is effective in improving social functioning for adolescents with ASD in Japan. Positive results were found specifically in the areas of socialization, communication, knowledge of social skills, autistic mannerisms, and behavioral and emotional problems. In addition, most treatment gains were maintained at a 3-month follow-up assessment. These findings suggest that the Japanese version of PEERS is beneficial across multiple socio-emotional and behavioral domains for adolescents with ASD.

Individuals with autism spectrum disorder (ASD) are characterized by deficits in social communication and social interaction, and restricted, repetitive patterns of behavior, interests, or activities (American Psychiatric Association [APA] 2013). Because of these characteristics, children and adolescents with ASD often experience difficulties in social adjustments such as making friends and maintaining good relationships. Because friendships are known to protect against bullying and contribute to positive emotional outcomes, developing meaningful friendships is in turn thought to improve quality of life and reduce the risk of mental health problems (Laugeson and Frankel 2010). Thus, it is critical to support the development of social skills for adolescents with ASD.

Difficulty in making friends and maintaining good relationships among youth with ASD has been demonstrated in numerous studies. One such study (Bauminger and Kasari 2000) reported that high-functioning children with ASD exhibited a different understanding of friendship compared with typically developing children. That study revealed that all of the children with ASD believed they had a friend, yet their perceptions of these friendships did not reflect mutual and secure relationships. Another study found that among children with ASD who were actively attempting to develop friendships, the likelihood of rejection and even bullying was greater compared with those who were not seeking friendships (Mazurek and Kanne 2010). According to the authors, this was most likely because of the lack of sufficient social skills needed to develop and maintain these relationships. More specifically, utilizing social skills for conversation, get-togethers, and problem solving would help them maintain relationships and become mutual friends with peers.

Given the plethora of findings highlighting the negative consequences of social impairments in youth with ASD (Howlin 2000; Locke et al. 2010; Mazurek and Kanne 2010; Sadolescentsel and Heeman 2017; White et al. 2010), the need for social skills training programs for adolescents with ASD is growing. Previous research has shown that participants with ASD who completed a social skills intervention focusing on friendships had significantly improved vocal expressiveness, as well as improved overall quality of rapport (Dolan et al. 2016). In addition, teaching skills through group-based interventions was found to be effective for children with high functioning ASD to develop comfort and confidence in social interactions (DeRosier et al. 2011; Tse et al. 2007). Although the number of treatment programs for youth with ASD is expected to increase further (Miller et al. 2014), most of the research addressing social skills treatment in autism continues to focus on children at earlier developmental ages, and there are still very few evidence-based interventions for autistic adolescents (White et al. 2007).

As for the development of children with ASD, adolescence is an important stage since this is when they become conscious of the difficulties in interacting with others (Tse et al. 2007). It is argued that friendship for typically developing children generally starts from having common activities and places, and as they enter adolescence, having common interests becomes more important (Nitto and Fujino 2017). In a developmental stage such as this, ASD adolescents, who tend to have poor social cognition, would face difficulties in detecting and sharing common interests with others, and this would result in a scarcity of peer relationships. Having one or two close friends during adolescence can predict later adjustment in life and can buffer the impact of stressful life events, which correlates positively with self-esteem and independence (Buhrmester 1990). Conversely, a lack of close friendships would not only influence the above factors negatively, but also lead to a more serious situation. High functioning adolescents with ASD appear to be especially at risk for developing anxiety disorders (Sadolescentsel and Heeman 2017) and behavioral and emotional problems (White et al. 2009a). To prevent these problems and help adolescents make a successful transition into adulthood, friendship support could be a predictor of resilient functioning in psychosocial domains later in life (Halmelen et al. 2017). Therefore, effective social skills training programs are strongly needed (Laugeson and Park 2014; Schohl et al. 2013; White et al. 2007).

Among various social skills training programs for people with ASD, the Program for the Education and Enrichment of Relational Skills (PEERS; Laugeson and Frankel 2010) is one of the few backed by scientific evidence. This program, which focuses on skills related to making friends and maintaining good relationships (Laugeson and Frankel 2010), has demonstrated not only immediate treatment gains for adolescents with ASD without intellectual disabilities (Dolan et al. 2016; Laugeson et al. 2009, 2012; Shum et al. 2018; Yoo et al. 2014), but also long-term treatment effects, even 1 to 5 years following the intervention (Mandelberg et al. 2014). In addition, although the PEERS intervention does not aim to reduce social anxiety, a previous study showed significant decreases in social anxiety symptoms as compared with the waitlist control group (Hill et al. 2017; Schohl et al. 2013). This finding is promising because adolescents may become more likely to interact with peers by learning social skills.

Several important features of the program are thought to lead to these positive results. First, evidence-based methods of instruction taken from the principles of cognitive behavioral treatment (CBT) are used to teach social skills in PEERS. This includes a small group format (eight to ten participants), didactic lessons utilizing Socratic questioning, role-play demonstrations, behavioral rehearsal exercises with performance feedback through coaching, and weekly socialization homework assignments. Socratic questioning is a common CBT method that involves a systematic line of questioning that guides reasoning. Through the discussion with a leader using this questioning style, participants are more likely to believe what they are learning (Laugeson and Park 2014). Whereas numerous concerns have been expressed toward existing social skills programs, those based on CBT are acknowledged as assuring treatment methods to alleviate social deficits in ASD adolescents (Laugeson and Park 2014). Second, PEERS is a parent-mediated intervention, which is a therapeutic treatment method known to be highly effective for improving the social skills of youth with ASD (Frankel et al. 2010; Smith et al. 2018). Parental assistance is promoted through concurrent weekly social coaching groups for parents, which are conducted to encourage the generalization of skills to more natural social settings and the maintenance of treatment gains over time (Laugeson and Park 2014). Third, each session of the program includes concrete rules and steps for ecologically valid social skills exhibited by typically developing adolescents through a manualized intervention that ensures treatment fidelity and replicability (Laugeson and Frankel 2010).

In a previous review (Miller et al. 2014), social skills group interventions were found to be effective in their countries of origin have also been tested in different countries and shown evidence for generalizability, even in different cultural contexts. PEERS was originally developed in the United States, and has been shown to be effective in multiple trials in North America (Dolan et al. 2016; Hill et al. 2017; Laugeson et al. 2009, 2012; Gantman et al. 2012; Schohl et al. 2013; Laugeson et al. 2014, 2015). PEERS has also been tested within Asia. Specifically, a Korean research group based out of Seoul National University conducted a cross-cultural validation trial of PEERS and found the intervention to be efficacious for Korean adolescents (Yoo et al. 2014). A Chinese research group based out of Hong Kong University also conducted a cross-cultural validation and found PEERS to be effective for Chinese adolescents (Shum et al. 2018), with only minor adaptations. While these findings are encouraging, PEERS has yet to be examined in other countries within Asia, including Japan. Even though Japan is also located in the East Asian region, each country has a different cultural background (Park et al. 2017), and thus, the cultural adaption of the program should be carefully examined. Therefore, evidence-based programs such as PEERS are strongly desired in Japan, and cross-cultural validation is very meaningful (Okajima and Suzuki 2012).

The purpose of the present study was to adapt the PEERS intervention for a new cultural context and to examine the effectiveness of PEERS in improving the social functioning of adolescents with ASD without intellectual disabilities in Japan. To conduct a cross-cultural validation trial of PEERS, we examined changes in social functioning across two groups: one that receives the treatment immediately (treatment group), and one that receives treatment following a waiting period (delayed treatment control group). Four hypotheses were tested: (1) adolescents who received the treatment immediately would show a significant positive change in social functioning compared with adolescents waiting for treatment; (2) the social skills of adolescents in both groups would improve immediately after treatment; (3) the positive effects of the treatment would be maintained for 14 weeks after treatment; and (4) the number of adolescents with clinically severe mental health conditions would decrease following the treatment.

## Methods

### Participants

In total, 28 elementary and middle school adolescents ranging in age from 11 to 15 years (*M *= 13.08; SD = 13.66) participated in the study along with their parents. All participants (19 males and 9 females) were born and raised in Japan, spoke fluent Japanese, had a parent willing and able to participate in the treatment, and had a previous diagnosis of ASD from a pediatrician specializing in developmental disabilities with more than 10 years of medical experience. The participants were confirmed based on the total score (*M *= 68.14; SD = 8.88) on the Japanese version of the Social Responsiveness Scale (SRS-2; Constantino 2009). For this study, the research team asked a parent to fill out the questionnaire at pre-treatment. Ten of the 28 participants were diagnosed via the Autism Diagnostic Observation Schedule (ADOS; Lord et al. 2012). Intellectual functioning was assessed using the Japanese version of the Wechsler Intelligence Scale for Children (WISC-III; Wechsler 1998 and WISC-IV; Wechsler 2010). Regarding the data from the WISC-III/IV and ADOS, past records were obtained at the time of recruitment. Participants whose WISC-III or WISC-IV data were collected more than 18 months prior to recruitment were assessed again to receive a new record at baseline. Treatment motivation was also a requirement for all participants. Prior to study inclusion, all participants were required to express their motivation to learn the skills related to friendships taught in PEERS and to agree to attend weekly 90-min social skills groups with their parents. Parents were also required to agree to attend all sessions to learn not only about the skills for making and keeping friends, but also how to help their children master those skills. In a previous study, those missing more than three out of 14 sessions were excluded from the sample (Laugeson et al. 2009). One participant in our study was absent for four sessions in our study because of competing school events. The researchers analyzed all participants including this participant since the mother attended the parent sessions and helped the adolescent learn the skills presented during the absences. The participants attended an average of 13.1 sessions (SD = 1.07). Overall, the adolescent attendance rate was 93.9%, as calculated by the total number of sessions attended as a proportion of the total number of sessions provided. All participants were treated in accordance with APA guidelines, received the treatment free of charge, and were permitted to withdraw from the study at any time according to their own decision and with no penalty. When an adolescent was absent, their parent was required to explain the skills learned during the parent sessions and to conduct behavior rehearsals with that adolescent. In addition, our study team advised the adolescent to arrive 10 min earlier than scheduled to take part in behavior rehearsals with a group leader and coaches. There was no attrition from either group, and the reasons given for the minimal absences were mostly school-related events as opposed to treatment resistance.

In addition, the overall homework completion rate was 82.3% for the sample, as measured by the percentage of total homework assigned compared with the total homework accomplished. The typical reason given for not completing a homework assignment was the lack of an opportunity to practice skills related to challenging situations such as teasing, bullying, and gossip. In such cases, the group leaders advised the students to review and practice the skills at home.

### Outcome Measures

To evaluate changes in social skills across the treatment and delayed treatment control groups, a total of eight outcome measures were collected. Six questionnaires—the SRS-2, Social Communication Questionnaire (SCQ), Test of Adolescent Social Skills Knowledge (TASSK), Quality of Play Questionnaire-Adolescent (QPQ-A), Quality of Play Questionnaire-Parent (QPQ-P), and Vineland Adaptive Behavior Scales, Second Edition (VABS-2)—were quantified as the primary end point, to evaluate main effect of the intervention, and two questionnaires—the Child Behavior Checklist (CBCL) and Depression Self-Rating Scale for Children (DSRS-C)—were quantified as the secondary end point, to evaluate additional effects of the intervention. Most of these measures were selected from previous PEERS studies (Laugeson et al. 2012, 2015; Mandelberg et al. 2014; Schohl et al. 2013; Yoo et al. 2014) because the research team considered that making a reference to those scores would be beneficial to assess the effect of the program in Japan. The SCQ, one of the primary measures, was previously used in a Korean study (Yoo et al. 2014). Most previous studies did not use the secondary outcome measures, except for the same Korean study (Yoo et al. 2014), or used a different one, the Social Interaction Anxiety Scale (Schohl et al. 2013). Our study team chose the CBCL and DSRS-C because emotional/behavioral problems in adolescents need to be assessed to understand possible mediating factors (Yoo et al. 2014) between anxiety and social deficit in ASD (White et al. 2010). Among these measures, the TASSK and QPQ-A/P, the SRS-2 and SCQ and the CBCL and DSRS-C are proximal measures. Adolescents answered three of the outcome measures—the TASSK, the DSRS-C and QPQ-A—and parents answered those remaining.

#### Primary Outcome Measures

##### Japanese Version of the Social Responsiveness Scale-2 (SRS-2; Constantino 2009)

The SRS-2 (Constantino 2009) is a 65-item parent-report scale that measures social impairments of children (4–18 years old), and has been confirmed as a reliable assessment tool for assessing autistic traits. This autism screening tool uses T-scores with a mean of 50 and a standard deviation of 10. Total scores ≥ 60 suggest clinical severity consistent with ASD symptomatology. The SRS-2 was selected as an outcome measure in the present study based on numerous reports demonstrating a positive change in scores on the SRS-2 following the implementation of PEERS (Laugeson et al. 2012; Gantman et al. 2012; Laugeson et al. 2015). Other published studies of social skills training or CBT for youth with ASD have also yielded positive results (DeRosier et al. 2011; Nakanishi et al. 2016; Tse et al. 2007; White et al. 2009a). The Japanese version of the SRS-2 is a published, standardized measure of social responsiveness (Moriwaki et al. 2011) widely used in the Japanese general child population, as well as in clinical settings. According to a survey of Japanese school children, autistic behavioral traits on the SRS-2 did not significantly differ from patterns observed in the United States or Europe, but traits measured quantitatively by parents differed somewhat according to culture (Kamio et al. 2013a). Therefore, ASD severity ratings should be carefully interpreted in consideration of cultural contexts.

##### Japanese Version of the Social Communication Questionnaire (SCQ; Rutter et al. 2013)

The SCQ is a 40-item, parent-completed questionnaire used to screen for autistic symptoms. The SCQ, which uses a yes/no response form, is based on the initial mandatory probes from the original Revised Autism Diagnostic Interview (Le Couteur et al. 2003). There are two different versions of the SCQ: a “lifetime” version, which is designed to assess children from birth, with questions #20 to #40 focused on the 12-month period of a child’s life between the ages of 4–5 years, and a “current” version, which is designed to assess the present condition of a child based on the past 3 months. The present study used the “lifetime” version as a descriptive measure at baseline and the “current” version as an outcome measure. Cutoff scores ≥ 15 on the SCQ suggest the presence of significant autism symptoms.

##### Japanese Version of the Test of Adolescent Social Skills Knowledge (TASSK; Laugeson and Frankel 2010)

The TASSK is a 26-item, criterion-based measure completed by adolescents to assess whether they understood the skills presented in PEERS (Laugeson and Frankel 2010). Two items are derived from each of the 13 lessons, with each question including buzzwords related to specific social skills knowledge taught during the intervention. Adolescents select one answer from two choices to match the skill they should use in a presented situation. Total scores range from 0 to 26, with higher scores indicating greater knowledge of targeted social skills. The English version was translated into Japanese by the research team, back-translated by professional translators, and then confirmed by Dr. Laugeson, a developer of the PEERS curriculum and this assessment, who is also the last author of the present study. The reliability index, the omega coefficient, was .75, .88, and .92 for the TASSK at each measurement point (pre-test, post-test, and follow-up test, respectively) in the present study. To compare these results with previous studies, Cronbach’s alpha coefficient was also calculated. The alpha coefficients and 95% confidence intervals for the TASSK were .53 [.29, .78], .87 [.80, .94], and .91 [.86, .96] at each measurement point, showing relatively low values, in the pre-test. Previous PEERS studies have shown that the TASSK is sensitive to a treatment effect, while low alpha coefficients have also been reported (Laugeson et al. 2009, 2012; Mandelberg et al. 2014; Schohl et al. 2013). For example, Laugeson et al. (2009) reported an alpha coefficient of 0.56 for this measure, and explained that this level of internal consistency was acceptable given the wide domain of questions on the scale because the questions were not expected to agree with one another.

##### Japanese Version of the Quality of Play Questionnaire (QPQ; Laugeson and Frankel 2010)

The QPQ consists of 12 items completed by parents and adolescents to assess the number of get-togethers, both hosted and invited, over the previous month (Laugeson and Frankel 2010). The QPQ also includes 10 items assessing conflict during the last hosted get-together. Higher scores on the Conflict Scale indicate more conflict during get-togethers. This assessment was created by the developers of PEERS to measure social engagement, can be found in the appendices of the published PEERS manual, and has been used as an outcome measure in most previous PEERS studies (Gantman et al. 2012; Laugeson et al. 2009, 2012, 2014, 2015; Mandelberg et al. 2014; Schohl et al. 2013; Yoo et al. 2014). The QPQ was slightly modified by the research team with the permission of a developer of PEERS, who is also the last author of the present study. With regard to modifications, the English version of the QPQ instructs participants to “Please indicate how many get-togethers you hosted in the last month” and “Please indicate how many get-togethers you attended at another adolescents’ house in the past month”. To adapt to Asian culture, the Japanese version of the QPQ also includes the item, “Please indicate how many get-togethers you attended outside of the house”, since the majority of get-togethers in Japan take place in the community rather than in the home. The English version was translated into Japanese by the research team, back-translated by professional translators, and then confirmed by Dr. Laugeson, a developer of the PEERS curriculum and this assessment, who is also the last author of the present study. The reliability index, the omega coefficient, for the QPQ-A was .75, .83, and .82 for the Conflict Scale at each measurement point (pre-test, post-test, and follow-up test, respectively) in the present study, while those for the QPQ-P were .56, .79, and .74, respectively. To compare these results with previous studies, Cronbach’s alpha coefficients were also calculated. The alpha coefficients and 95% confidence intervals for the Conflict Scale on the QPQ-A were .74 [.59, .88], .80 [.69, .91], and .81 [.70, .91] at each measurement point, while those for the Conflict Scale on the QPQ-P were .36 [.00, .71], .78 [.65, .90], and .69 [.53, .86].

##### Japanese Version of the Vineland Adaptive Behavior Scales, Second Edition (VABS-2; Sparrow et al. 2014)

The VABS-2 is a measure of adaptive behavioral skills administered through semi-structured interviews with parents to assess functioning in the following main domains: communication, daily living skills, socialization, and maladaptive behavior index (Sparrow et al. 2014). The content validity and reliability of the Japanese version of the VABS-2 has been established with a reliability coefficient for the Japanese Adaptive Behavior composite score of .81 (Kamio et al. 2013b). Although the VABS-2 has only been used as a demographic variable in previous PEERS studies (Gantman et al. 2012; Laugeson et al. 2009, 2012), it was used both as a demographic variable and an outcome measure in the present study. The VABS-2 uses standard scores with a mean of 100 and a standard deviation of 15, with scores ≤ 70 points in the “low” range of adaptive functioning, suggesting clinical severity.

#### Secondary Outcome Measures

##### Japanese Version of the Child Behavior Checklist (CBCL; Achenbach 2001)

The CBCL/4–18 is a parent-reported scale commonly used for clinical and research purposes to measure behavioral and emotional problems in children (Achenbach 2001). The first section of the CBCL asks the parent to rate the child’s social competencies, such as participation in sports, hobbies, social activities, chores, social interactions with friends and family members, and academic performance. The second section consists of 120 items on behavior or emotional problems during the past 6 months as rated on a three-point scale. The main areas of this construct are withdrawal, somatic complaints, anxious/depressed, social problems, thought problems, attention problems, delinquent behavior, and aggressive behavior. The Japanese version of the CBCL/4–18 was standardized in 2001 and has a cutoff T-score of 63.

##### Japanese Version of Depression Self-rating Scale for Children (DSRS-C; Birleson 2011)

The DSRS-C is an 18-item self-report measure assessing depressive symptoms in children (Birleson 2011). Each item is rated on a three-point Likert scale as follows: 0 (never), 1 (sometimes), and 2 (always). Standardization of the Japanese version of the DSRS-C has shown sufficient reliability and validity, with average scores of 19.3 for the depressive group and 10.7 for the non-depressive group, and 16 as the clinical cutoff score.

### Procedure

Participants were recruited from a university hospital and one school district within Osaka, Japan, with referrals from pediatric doctors and clinical psychologists. Informed consent was obtained from all parents and adolescents who participated in this study, and all research was conducted with approval from the ethics committees of Kanazawa University and Osaka University.

The eligibility criteria for the adolescents were as follows: (a) age 11–15 years; (b) currently enrolled in school between the sixth grade of elementary school to the third grade of middle school; (c) a previous diagnosis from a reliable mental health or medical professional as having either ASD, high-functioning autism, Asperger’s syndrome, pervasive developmental disorder, or pervasive developmental disorder-not otherwise specified; (d) a verbal intelligence quotient (IQ) > 70 as determined by the WISC-III (Wechsler 1998) or WISC-IV (Wechsler 2010); (e) willingness to participate in the study expressed at the interview for recruitment (f) no history of major mental illness reported by a parent, including bipolar disorder, schizophrenia, or psychosis; (g) no current disruptive behavioral problems, aggression, or severe oppositional attitude; and (h) no auditory, visual, or physical impairments that would prevent participation in outdoor sports activities.

**Fig. 1:**
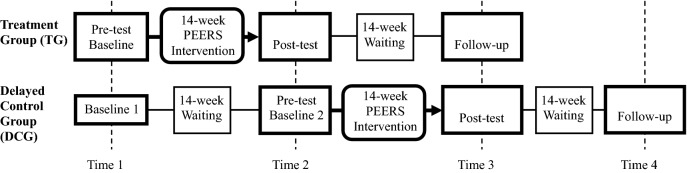
Research design

Eligible participants were assigned to one of two groups: one that received the treatment immediately (treatment group; TG), or one that received treatment following a 14-week waiting period (delayed treatment control group; DCG). Participants in this study were assigned to one of the two groups according to their school schedule; thus, randomization was not possible. The participants in the TG (*n *= 14) were assessed at baseline (Time 1: pre-test), started to receive the 14-week treatment immediately, were assessed just after the treatment was completed (Time 2: post-test), and returned for a follow-up assessment 14 weeks after the end of the treatment (Time 3: follow-up). Participants in the DCG (*n *= 14) were assessed at baseline (Time 1: baseline 1), waited for 14 weeks before receiving the treatment, received a second baseline assessment 1 week prior to the first session (Time 2: baseline 2), a post-assessment just after the treatment (Time 3: post-test), and then returned for a follow-up assessment 14 weeks after the treatment (Time 4: follow-up). Therefore, the TG underwent three assessments and the DCG four. Figure 1 provides an overview of the research design. At the end of the program, comments and feedback were informally obtained from adolescents and parents.

Adolescents and parents attended each session separately, but concurrently in different rooms. For both the parents’ and adolescents’ sessions, two TG and DCG each were run, with each group having seven participants. Although randomization was not used for the group assignments, no discernable differences were observed between the TG and DCG at the baseline, except for the TASSK scores. The profile of each group is described in Table 2. The adolescent group had one group leader and 1–3 behavioral coaches (mean = 1.6, SD = 0.69) who assisted the session by conducting role-play demonstrations, providing performance feedback through coaching during the behavioral rehearsal activities, providing behavioral management when necessary, and monitoring treatment fidelity to ensure that all aspects of the program were covered in their entirety. The adolescent group leader for both the TG and DCG cohorts was the primary author of this paper, a licensed clinical psychologist with more than 10 years of experience working with adolescents with ASD, and is a certified PEERS provider, having received 24 h of training by the program developer, Dr. Laugeson, through the UCLA PEERS Clinic. Behavioral coaches in the adolescent sessions consisted of graduate school students who were majoring in clinical or developmental psychology, had several years of experience working with children with developmental disorders, including ASD, and were trained and supervised by the adolescent group leader. Two parent group leaders were responsible for leading the weekly parent sessions: one was a clinical developmental psychologist, and the other was a graduate student specialized in developmental psychology who had experience working as a special education teacher at a public school for several years. Both parent leaders also had substantial experience working with children and adolescents with ASD.

#### Overview of the PEERS Curriculum and Japanese Cultural Adaptations

The English version of the PEERS Treatment Manual (Laugeson and Frankel 2010) was translated into Japanese by three members of the research team who specialize in the social development of children with ASD. To maintain consistency across the curriculum, two other research members, who were not the therapists for this intervention, checked the details to ensure that the original curriculum contents were included in the Japanese version. For example, they checked the consistent use of the same buzzwords and perspective questions after each role-play demonstration. The original PEERS curriculum was unchanged, except for minor adjustments related to cultural and social environmental differences between the United States and Japan, which are discussed below.

To examine which parts of the Japanese version needed to be adjusted, the authors received feedback from the participants during the trial sessions practiced prior to the experiment. The participants in the trial group were two adolescents and their parents. One research team member led an adolescent and a parent session. Following the PEERS manual, a weekly 90-min session was held at an educational center for 14 weeks. The session leader observed and checked how these participants responded to the curriculum. Then, the research team members discussed if anything needed to be changed. Through that process, we decided to change several terms and various contents, as listed in Table 1. Other than these minor changes, most of the structure and instructions of the original manual were maintained.

**Table 1:** Overview of the PEERS curriculum and Japanese cultural adaptations

Session	Didactic lesson	Description of the lesson^a^	Cultural issues	Rationale for cultural modification
1	Introduction and conversational skills I: trading information	How to trade information during conversations with peers to find common interests	(T) Jeopardy topics: “The Eyes Have It” and “School Spirit”	Japanese eye color is generally similar, so “The Eyes Have it” topic was removed from Jeopardy. Japanese students are not familiar with the term “school spirit,” so this topic was replaced in Jeopardy with favorite subject and school name
2	Conversational skills II: two-way conversations	The key elements of having a two-way conversation with peers (P) Beginning to identify adolescent activities that could lead to potential sources for friendships	(P) Sources of Friends	Additional sources of friends were included to be relevant to Japanese culture: card games, online games, *otaku* (someone who loves specific culture), history girl, dance club, *shogi* (Japanese chess), *igo*, Japanese traditional sports (judo, karate, kendo), and cosplay
3	Conversational skills III: electronic communication	The appropriate use of voice mail, e-mail, text messaging, instant messaging, and the Internet in developing preexisting friendships further	Facebook/MySpace	Facebook is popular in Japanese culture, but not among adolescents, and MySpace no longer exists in Japan, so both were removed from electronic communication. For children and adolescents in Japan, Line is a more common SNS for their communication, so was added to this lesson
			(P) Different Peer Group or Crowds	Some groups on this list are not common for Japanese adolescents. Thus, the same table (session 2) was used for this session
4	Choosing appropriate friends	(T) Identifying peer groups they might fit in with and identifying extracurricular activities based on the adolescent interests, which might lead to new sources of friends with common interests	(T) Different Peer Group or Crowds	Some peer groups in North America are not common for Japanese adolescents, so several groups were deleted and additional sources of friends were included (the same mentioned in parent session 2)
			(P) Sources of Friends	The same additional sources of friends that were mentioned in parent session 2 were included
5	Appropriate use of humor	The basic rules around the appropriate use of humor	(T) Knock-knock joke used in the role-play and behavioral rehearsal	The English manual uses a common knock–knock joke during the role-play and behavioral rehearsal exercises to teach skills related to paying attention to humor feedback. A common Japanese joke was used in this lesson to replace the English knock–knock joke
6	Peer entry I: entering a conversation	The steps involved in joining conversations with peers	Good and bad places to make friends	Places uncommon for Japanese adolescents to make new friends were deleted (i.e., school bus and community pool)
7	Peer entry II: Exiting a conversation	How to assess receptiveness during peer entry and how to gracefully exit conversations when you are not accepted	N/A	No cultural adaptations were made to this lesson
8	Get-togethers	How to plan and implement successful get-togethers with friends	Suggestions for activities during get-togethers	Some suggested activities from the original manual had to be changed to fit the cultural context for Japanese adolescents (i.e., removal of barbecues, miniature golf, water parks, dog parks, playing pool, laser tag; addition of karaoke)
			Steps for beginning get-togethers at your home	Giving guests a tour of one’s home is not a Japanese custom for adolescents. Only the living room and bathroom are shown to guests. Also, adolescents do not typically ask their guests what they would like to eat or drink; however, this step was retained as a formal way to welcome guests
9	Good sportsmanship	The rules of good sportsmanship	N/A	No cultural adaptations were made to this lesson
10	Rejection I: Teasing and Embarrassing Feedback	How to appropriately respond to teasing from peers/how to differentiate between teasing (i.e., verbal attacks) and embarrassing feedback, and how to alter their behavior in response to the latter	Tactics for showing that you do not care about the teasing	Verbal responses that demonstrate they do not care (i.e., shrugging shoulders and rolling eyes) are not natural expressions for Japanese youth. Only short verbal comebacks, followed by walking away, were retained
			Brief comebacks used to make fun of what the person said	Adolescents chose from a list of short verbal responses deemed appropriate in Japanese culture. Japanese responses were the equivalent of the English version of verbal comebacks such as, “So what?”, “Who cares?”, and “Yeah, and?”
11	Rejection II: Bullying and bad reputations	The strategies for handling bullying (i.e., physical attacks) and how to change bad reputations	N/A	No cultural adaptations were made to this lesson
12	Handling disagreements	The important elements necessary to resolve arguments and disagreements with peers	Saying you are sorry	Consistent with the original PEERS manual, it is helpful to say you are sorry when someone is angry, sad, or upset, because the person is feeling bad and wants you to acknowledge that you are sorry that he or she is feeling that way. To make this point clear for Japanese adolescents, the statement “I’m sorry *if* you feel that way” was added, consistent with the Korean cultural adaptations described in Yoo et al. (2014)
13	Rumors and gossip	The strategies for minimizing the effects of rumors and gossip	N/A	No cultural adaptations were made to this lesson
14	Graduation and termination	A graduation party and a ceremony (P) the strategies to maintain gains in adolescent social skills after termination	N/A	No cultural adaptations were made to this lesson

(T) content only for adolescents’ session

(P) content only for parents’ session

^a^Description of the lesson (Laugeson et al. 2012) (Laugeson and Frankel 2010)

To clarify the current situation regarding the get-togethers, the teasing behaviors, and after-school activities in Japan, 12 questions were posed to 258 middle-school students in Osaka, Japan. The survey questions were related to how often they play with friends, where they play, what they usually do with their friends, what they do after school other than get-togethers, and teasing behaviors. According to the results, we revised the terms in sessions 2, 4, and 8.

Another culturally sensitive issue is related to the “Appropriate use of humor” (session 5). As an example, team members collected Japanese jokes for children and discussed which one would be appropriate for this session. Besides these issues, in the process of translation, clinical psychologists, clinical developmental psychologists, a Japanese language teacher at a middle school, and the supervisors of this study repeatedly discussed if each didactic element was appropriate for Japanese adolescents. Modifications including the above items are listed in Table 1.

PEERS consists of 90-min sessions offered once a week over the course of 14 weeks (Laugeson and Frankel 2010). The first 30 min of the adolescent session begin by reviewing homework assigned in the previous week. The next 30 min include a didactic lesson with role-play demonstrations targeting specific social skills related to making and keeping friends or handling peer conflict and rejection. Within the role-play demonstration, adolescents observe appropriate and inappropriate social behavior performed by the adolescent group leader and behavioral coaches. The next 20 min are spent with the adolescents practicing the skills they just learned through behavioral rehearsal activities, including structured games, while receiving performance feedback from the group leader and behavioral coaches. The remaining 10 min are spent reunifying with parents to briefly discuss what the adolescents learned and negotiate how they will practice the newly learned skills during the week through parent-assisted socialization homework assignments. Each 90-min parent session also begins with a homework review in which families discuss problems arising during homework exercises while the group leader troubleshoots any outstanding issues and provides social coaching tips. Since the homework review is a core component of the parent session, two-thirds of the session time (approximately 60 min) is spent on this discussion. Within the next 20 min, parents receive a didactic lesson with a social coaching handout outlining the adolescents’ lesson, are assigned homework for the next week, and receive instruction on how to help their child utilize the skills. Finally, as previously mentioned, at the end of each session is a 10-min reunification time in which the adolescents join their parents and review the new skills they have learned while discussing the upcoming homework assignments and individually negotiating how to ensure they are completed.

The PEERS approach applies CBT instruction methods, including didactic lessons (psychoeducation), role-play demonstrations, cognitive strategies, behavioral rehearsal exercises, performance feedback, homework assignments and review, and parent involvement within a small group treatment format (approximately seven members per group). Including parents as an essential element of the treatment is one of the distinctive features of the PEERS method (Laugeson and Park 2014). Parents concurrently learn each targeted social skill and know how to assist their adolescents in mastering those skills in natural social settings. Parents are an integral part of ensuring that adolescents practice homework assignments in real world settings and can provide assistance in challenging real-world situations. Since parents often observe their adolescents practicing newly learned skills, they can give practical advice based on the PEERS curriculum through their social coaching. The parents’ role as a social coach is essential for adolescents to become successful in this program, and is thought to lead to greater skills generalization. Moreover, by including parents as social coaches, the notion is that the program never ends, thereby enhancing the durability of treatment gains over time.

An overview of the PEERS curriculum is provided in Table 1. Apart from the linguistic adaptation that occurs with any translated work, the Japanese version of PEERS basically follows the original North American curriculum, since most of the rules and steps of the social skills presented in the curriculum are fairly applicable in the Japanese cultural setting, which has become quite Westernized. However, since there are always cultural differences in expected behaviors across society (Miura 2013), minor changes were introduced. For example, in Session 1, the eye color category in the Jeopardy game in which adolescents are instructed to make eye contact to determine the eye color of their teammates, was not suitable for Japanese adolescents. Similar to the adaptations made to the Korean version of PEERS (Yoo et al. 2014), most Japanese people have very similar eye colors, so this category was deleted. In addition, regarding another category in this game, Japanese teachers do not often use the term “school spirit” to describe the characteristics of their school, which means that students are not generally familiar with the term. Instead, other categories were used in this study, such as favorite school subject.

In Sessions 2, 3, 4 and 6, we added several places and groups currently popular in Japanese culture to the sources of potential friends, as described in Table 1. In Session 3, regarding popular online social networking sites, “Line” was included in the curriculum because it is a widely used service for electronic communication among adolescents in Japan.

In Session 8, where the focus of the lesson is on having get-togethers with friends, the Japanese curriculum was modified to include only a brief tour of one’s home (i.e., two relevant rooms instead of the entire house), since it is uncommon to give guests a tour of one’s entire home. The living room and a bathroom were chosen because being familiar with these rooms could help guests be comfortable while spending time at a friend’s house. However, in reality, many get-togethers are not spent in one’s home, but in one’s community. Another survey found that approximately 35% of fifth and eighth graders play with friends outside of their homes, while only 16% play with friends inside their homes (Kashiwa City Board of Education and Kawamura Gakuen Woman’s University 2010). Thus, community-based get-togethers may be more typical among Japanese youths.

Perhaps the most profound change to the PEERS curriculum also relates to the session on get-togethers and how Japanese adolescents spend their time after school. According to a survey of Japanese youths (Benesse Educational Research and Development Institute 2013), approximately 92% of seventh graders and 90% of eighth graders join in after-school clubs and activities offered by their school, and about 60% of these youth continue participation in such clubs during weekends. This high level of engagement in after-school activities may leave little time for additional get-togethers with friends. Moreover, consistent with other Asian cultures, because of the strong emphasis on academics and school entrance exams in Japan, adolescents typically have less time to play after school and fewer get-togethers. One recent estimate suggested that approximately 64% of ninth graders attend an after-school class or “cram school” to prepare for exams, and more than 60% of these students attend classes three times a week (Benesse Educational Research and Development Institute 2013). According to participants in the present study, when adolescents want to socialize with their friends in the context of such high academic pressure, they typically interact for a short period of time on their way home from club activities or cram schools. Therefore, the importance of having regular get-togethers with one’s friends outside of school may need to be reconsidered within the cultural framework of Japanese life for youths.

Finally, with regard to cultural modifications, in Session 10, adolescents learn how to respond to verbal bullying or teasing. Unlike in Northern America, shrugging one’s shoulders and rolling one’s eyes is not common body language in Japan. Therefore, nonverbal comebacks were entirely replaced by verbal comebacks such as “So what?” or “Whatever”. Since culture plays an important role in forming nonverbal behaviors (Matsumoto 2006), it is not surprising that indeed, adolescents in the present study indicated that short verbal comebacks were more comfortable for them.

The above treatment modifications, among the others highlighted in Table 1, have been made to adapt and account for cultural differences in the Japanese linguistic and cultural translation of the PEERS intervention manual.

#### Treatment Fidelity

Each week, during a clinical case conference meeting with parent group leaders and coaches, the adolescent group leader provided didactic training regarding the content of the forthcoming lesson. Team members also met weekly after each group to review what had happened during the session and to troubleshoot any clinical issues that may have arisen. As an effectiveness trial, achieving treatment fidelity and consistency with the original treatment manual was very important. Therefore, all staff members were trained on the protocol, reviewed the treatment manual thoroughly before each session, and were given didactic instruction prior to each session to ensure adequate understanding of the treatment content and methods. In addition, clinical supervisors and members of the research team, who were specialized in the development of children with ASD and familiar with the PEERS curriculum, monitored treatment fidelity by viewing videotapes of each session. Although quantitative data on treatment fidelity had not been collected, the videotape of each session was viewed by two supervisors every week. The validity of this study would have been stronger if we had any quantitative data to measure the treatment integrity.

#### Data Analysis

Four sets of analyses were conducted to evaluate the effectiveness of PEERS in Japan. Prior to testing the treatment effectiveness, an independent-samples *t* test and a Chi squared test were conducted to determine if there were any significant differences in demographic variables between the TG and DCG. Next, to evaluate the treatment effects across the two groups, a 2 (TG, DCG) × 2 (Time 1, Time 2) mixed-design analysis of variance (ANOVA) was used to examine the first hypothesis, that only the TG would show improvement at Time 2 while the DCG waited without receiving the treatment. To test the second hypothesis, that adolescents in both the TG and DCG would show improved social skills following treatment, another mixed ANOVA for the TG and DCG was used to evaluate the treatment effects (Time 1 and Time 2 for the TG; Time 2 and Time 3 for the DCG). To assess changes in social engagement as measured using the frequency of get-togethers in the previous month, a Wilcoxon signed-rank test was performed combining data from TG and DCG. To assess the third hypothesis, that a 3-month maintenance effect would be observed following treatment, a mixed ANOVA for the TG and DCG was performed examining pre-test and follow-up assessments. Also, a Wilcoxon signed-rank test was used for the analysis of frequency of get-togethers combining data from TG and DCG. Lastly, the fourth hypothesis, regarding the differences in the proportions of adolescents with and without clinically severe mental health conditions before and after treatment, was investigated using McNemar’s test. For the fourth hypotheses, the data for the two groups were combined. All statistical analyses were performed using SPSS version 23 (IBM Corp, Armonk, NY, USA).

## Results

Demographic and baseline variables were compared between the TG and DCG prior to treatment (Time 1). Independent-sample *t* tests between the two groups revealed no significant differences in age or IQ, and all outcome measures were equivalent, except for the TASSK scores. As for the gender ratio, 35.7% of TG members and 28.6% of DCG members were female. A Chi squared test of independence showed no significant difference in the gender ratio between groups (*χ*^2^ (1, *N *= 28) = 0.164, *p *= .69). Table 2 displays the comparisons of demographic and baseline variables between TG and DCG. Since the assumption of normal distribution was violated, using an independent t-test was considered inappropriate to assess the difference between TG and DCG in the QPQ-A/P Total get-togethers. Thus the Mann–Whitney test was used and showed there was no significant difference between two groups for QPQ-A (*U *= 77.5, *p *= .35) and QPQ-P (*U *= 83.0, *p *= .51).

**Table 2:** Comparisons of demographic and baseline variables between treatment group and delayed control group

Variable	Group		*t*	*p*
	Treatment (*n *= 14)	Delayed control (*n *= 14)		
	M (SD)	M (SD)		
Age (years)	13.00 (1.26)	13.17 (1.04)	− 0.39	.70
WISC-IV				
Full-scale IQ	103.36 (16.29)	107.43 (14.82)	− 0.69	.91
Adolescent measures				
TASSK	15.43 (2.76)	12.85 (3.54)	2.14	< .05*
DSRS-C	13.07 (6.18)	14.28 (3.93)	− 0.62	.54
Parent measures				
SRS-2	67.64 (7.40)	68.64 (10.40)	− 0.29	.77
SCQ				
Lifetime	11.21 (4.61)	13.92 (8.25)	− 1.07	.30
VABS-2				
Composite	55.07 (12.35)	45.07 (17.66)	1.74	.10
Communication	59.64 (19.58)	46.00 (19.02)	1.87	.07^†^
Socialization	54.21 (54.21)	46.57 (17.79)	1.33	.20
Maladaptive behavior	21.00 (0.88)	20.71 (1.54)	0.60	.55
CBCL	70.21 (8.97)	65.43 (16.46)	1.62	.12

The Mann–Whitney test was used to assess changes in the QPQ-A/P Total get-togethers (the results are presented in the main text)

*p < .05; ^†^*p *< .10

^a^QPQ adolescents/parents total: the total number of get-togethers adolescents attended inside and outside of the home in the past month

Descriptive statistics of the outcome measures for the TG and DCG are presented in Table 3. Subscales of the measures (SRS-2, VABS-2, and CBCL) were analyzed for comparison with previous studies and to investigate which sub-categories differed between pre- and post-treatment. To evaluate the changes in scores for the TG compared with the DCG between Time 1 (pre-test 1 for TG, pre-test 1 for DCG) and Time 2 (post-test for TG, pre-test 2 for DCG) a two-way mixed ANOVA was performed for each outcome measure (Table 4). Since multiplicity adjustment should be considered, the Bonferroni–Holm correction was applied to the subscales. According to this analysis, four measures (TASSK, VABS-2 composite, VABS-2 socialization, and VABS-2 play and leisure time) showed an interaction of the two variables (group and time) and a main effect of time. Before this assessment, we examined whether the assumptions for the ANOVAs had been met. Among all the measures, only VABS-2 maladaptive behavior did not satisfy the assumption of normal distribution; therefore, a non-parametric test (the Friedman test) was performed (*p *= .317). However, as the test did not reject the null hypothesis, no significant difference between TG and DCG on VABS-2 maladaptive behavior was evident.

**Table 3:** Descriptive statistics for treatment group and delayed control group

Variable	Group						
	Treatment group (TG)			Delayed control group (DCG)			
	Pre-test	Post-test	Follow-up	Pre-test 1	Pre-test 2	Post-test	Follow-up
	Time 1	Time 2	Time 3	Time 1	Time 2	Time 3	Time 4
	M (SD)	M (SD)	M (SD)	M (SD)	M (SD)	M (SD)	M (SD)
Adolescent measures							
TASSK^a^	15.43 (2.77)	21.64 (3.75)	21.29 (4.60)	12.85 (3.55)	15.14 (2.98)	21.71 (3.20)	20.36 (4.40)
DSRS-C	13.07 (6.18)	13.00 (6.87)	11.79 (5.85)	14.29 (3.93)	14.50 (5.27)	13.00 (4.76)	12.36 (4.27)
QPQ-adolescents Total get-togethers^a^	4.07 (6.30)	6.21 (9.25)	4.29 (5.88)	1.00 (2.04)	1.41 (2.77)	4.57 (5.26)	4.29 (4.98)
Conflict	1.38 (1.61)	1.69 (2.56)	1.92 (1.93)	3.57 (4.03)	3.72 (4.05)	2.50 (3.53)	2.69 (3.68)
Parent measures							
SRS-2 total	67.64 (7.41)	63.64 (9.34)	63.36 (11.63)	68.64 (10.40)	67.79 (8.79)	62.86 (10.86)	60.93 (10.10)
Social awareness	60.93 (11.18)	56.86 (8.07)	57.29 (10.99)	65.71 (12,69)	64.43 (11.76)	58.14 (13.35)	57.07 (12.57)
Social cognition	68.79 (9.38)	64.00 (10.32)	64.50 (13.60)	68.43 (6.63)	67.36 (8.99)	66.29 (9.65)	61.57 (6.24)
Social communication	70.14 (9.16)	66.93 (13.76)	64.29 (11.34)	68.50 (11.37)	69.07 (9.59)	62.57 (9.09)	61.36 (12.38)
Social motivation	59.36 (10.32)	58.64 (8.54)	58.71 (10.76)	61.07 (10.59)	60.93 (8.74)	57.86 (9.89)	57.93 (9.84)
Autistic mannerisms	64.93 (7.33)	60.64 (9.68)	60.86 (15.28)	66.07 (9.87)	63.21 (8.34)	59.43 (11.12)	58.71 (8.71)
SCQ Current	8.07 (4.65)	6.93 (4.76)	5.07 (5.43)	7.71 (5.36)	8.29 (5.73)	7.43 (6.26)	6.00 (6.28)
VABS-2 Composite^a^	55.07 (12.36)	65.79 (12.00)	78.00 (13.14)	45.07 (17.66)	49.00 (17.87)	68.21 (19.03)	75.93 (21,86)
Communication^a^	59.64 (19.58)	68.79 (17.76)	83.21 (18.60)	46.00 (19.02)	53.43 (18.42)	71.21 (18.13)	82.00 (20.46)
Socialization^a^	54.21 (12.20)	69.21 (11.96)	80.07 (9.83)	46.57 (17.75)	52.00 (18.47)	69.57 (16.75)	77.00 (15.86)
Interpersonal relationships^a^	58.42 (6.11)	65.07 (5.94)	68.71 (3.58)	51.93 (11.42)	55.71 (11.10)	65.93 (5.95)	67.29 (6.07)
Play and leisure time^a^	48.71 (4.03)	53.36 (3.10)	54.79 (3.68)	45.86 (7.89)	47.21 (7.88)	52.50 (5.32)	54.00 (3.68)
Coping skills^a^	40.86 (7.12)	47.93 (6.07)	53.14 (4.59)	34.29 (11.55)	37.71 (11.47)	48.43 (10.67)	51.57 (11.45)
Maladaptive behavior	21.00 (0.88)	19.57 (1.16)	18.79 (1.72)	20.71 (1.54)	20.50 (1.34)	18.79 (1.93)	17.64 (2.76)
CBCL total	70.21 (8.97)	67.14 (9.29)	66.71 (11.03)	65.43 (6.47)	64.42 (5.26)	60.71 (6.10)	60.43 (5.03)
Withdrawal	57.36 (6.34)	56.76 (5.12)	57.21 (5.75)	56.29 (5.54)	55.50 (5.77)	53.79 (6.19)	53.43 (5.14)
Somatization	52.79 (5.12)	51.71 (4.46)	51.29 (4.60)	50.50 (4.29)	50.79 (5.06)	49.36 (4.13)	49.21 (4.02)
Anxiety/depression	61.57 (7.86)	61.00 (6.42)	60.07 (9.02)	57.36 (7.58)	57.86 (6.22)	54.71 (5.51)	55.00 (5.67)
Social problems	72.21 (7.97)	70.07 (10.84)	68.79 (9.87)	68.86 (5.07)	67.57 (5.29)	65.93 (6.22)	65.79 (4.81)
Thought problems	63.00 (10.19)	56.57 (9.17)	59.85 (10.63)	58.00 (10.37)	57.14 (9.13)	53.50 (5.33)	53.14 (5.59)
Inattention	67.71 (10.25)	65.50 (9.83)	63.71 (10.90)	66.79 (5.22)	66.71 (7.35)	63.00 (6.06)	63.71 (5.62)
Delinquent behavior	54.21 (6.92)	53.29 (7.08)	53.99 (7.84)	52.93 (6.99)	53.14 (7.01)	51.00 (7.69)	50.78 (6.58)
Aggressive behavior	64.93 (11.63)	64.36 (12.83)	63.57 (11.88)	60.86 (7.80)	59.14 (6.65)	55.29 (5.86)	52.79 (13.68)
Internalizing problems	68.36 (9.61)	68.36 (9.61)	64.86 (11.91)	62.14 (7.07)	61.64 (6.18)	58.71 (6.10)	57.93 (6.37)
Externalizing problems	65.29 (12.68)	63.50 (13.39)	62.93 (12.72)	60.86 (9.14)	60.43 (8.55)	54.71 (8.58)	56.07 (6.86)
QPQ-parents							
Total get-togethers^a^	3.07 (5.73)	5.29 (7.01)	4.29 (5.30)	1.21 (1.63)	1.71 (3.47)	3.71 (4.03)	3.64 (5.14)
Conflict	3.33 (2.53)	4.14 (3.83)	2.83 (2.12)	5.14 (3.67)	4.10 (2.81)	2.71 (2.02)	3.17 (2.89)

^a^Higher scores indicate increased ability

**Table 4:** Results of the 2 (TG, DCG) × 2 (Time 1, Time 2) ANOVA

Variable	Group × time		*η*_*p*_^2^
	*F(1)*	*p*	
Adolescent measures			
TASSK	11.94	.002**	.32
DSRS-C	0.03	.857	.00
Parent measures			
SRS-2 total	2.28	.143	.08
Social awareness	0.71	.407	.03
Social cognition	1.22	.279	.05
Social communication	1.26	.272	.05
Social motivation	0.06	.817	.00
Autistic mannerisms	0.30	.589	.01
SCQ current	1.90	.180	.07
VABS-2 composite	9.5	.005**	.27
Communication	0.30	.588	.01
Socialization	18.42	.000*	.42
Interpersonal relationships	2.77	.108	.10
Play and leisure time	15.07	.001*	.37
Coping skills	5.86	.023	.18
Maladaptive behavior	9.66	.005*	.27
CBCL total	0.85	.365	.03
Withdrawal	0.01	.923	.00
Somatization	1.95	.174	.07
Anxiety/depression	0.32	.575	.01
Social problems	0.15	.706	.01
Thought problems	2.03	.166	.07
Inattention	0.57	.456	.02
Delinquent behavior	0.71	.407	.03
Aggressive behavior	0.26	.614	.01
Internalizing problems	0.09	.769	.00
Externalizing problems	0.29	.594	.01

The Wilcoxon signed-rank test was used to assess changes in the QPQ-A/P total get-togethers (the results are presented in the main text)

For the subscales, statistical significance was tested based on adjusted p-values calculated using the Bonferroni–Holm correction. Therefore, asterisks might not be shown, even with a p-value < .05

*p < .05; **p < .01; ***p < .001

For the following three other measures, no significant interactions were found, but the main effect of time was significant: SRS-2 (*F*(1,26) = 5.45, *p *< .05); VABS-2 communication (*F*(1,26) = 28.12, *p *< .001); and VABS-2 interpersonal relationships (*F*(1,26) = 36.96, *p *< .001). Among these, a post hoc analysis revealed that SRS-2 showed positive changes in the TG (*p *< .05), but not in the DCG. The other two measures, VABS-2 communication and VABS-2 interpersonal relationships, showed significant changes in both the TG (*p *< .001) and the DCG (*p *< .01). The remaining measures (SCQ-current and DSRS-C) showed no significant interactions or main effect of time, and post hoc analyses did not show a significant change in time in either group.

Next, two-way mixed ANOVAs were conducted to examine the changes in outcome measures between pre- and post-treatment and between pre-treatment and follow-up for the TG and DCG (Table 5). The pre-test 2 score was used for DCG as the pre-treatment score for the second, third and fourth hypotheses. Treatment effects were also analyzed for all the participants who completed the study. Before that, we conducted ANOVAs to examine whether the assumptions had been met. Among all measures, since the assumption of normal distribution was violated for four measures (TASSK, SCQ-current, VABS-2 maladaptive behavior, and CBCL-total), a non-parametric test (the Friedman test) was performed; all were rejected under the null hypothesis (TASSK, *p *< .001; SCQ-current, *p *< .001; VABS-2 maladaptive behavior, *p *< .001; and CBCL-total, *p *< .01). The results of the ANOVAs showed a main effect of time between pre- and post-test, and between pre-test and follow-up, for 13 outcome measures. For four measures (DSRS-C, SRS-2 social cognition, SCQ-current, and CBCL inattention), there was no main effect of time between pre- and post-test, but there was a main effect of time between pre-test and follow-up.

**Table 5:** Results of the 2 (TG, DCG) × 2 (pre–post, pre-follow-up) ANOVA

Variable	Pre-test to Post-test		*η*_*p*_^2^	Pre-test to Follow-up		*η*_*p*_^2^
	*F(1)*	*p*		*F(1)*	*p*	
Adolescent measures:						
TASSK	78.34	.000***	.75	39.83	.000***	.61
DSRS-C	1.37	.253	.05	4.45	.045*	.15
Parent measures						
SRS-2 total	19.37	.000***	.43	15.60	.001**	.38
Social awareness	11.09	.003*	.30	6.30	.019*	.20
Social cognition	3.68	.066^†^	.12	7.42	.011*	.22
Social communication	10.14	.004*	.28	17.15	.000*	.40
Social motivation	2.47	.128	.09	1.56	.224	.06
Autistic mannerisms	9.81	.004*	.27	6.79	.015*	.21
SCQ current	2.62	.117	.09	15.56	.001**	.37
VABS-2 Composite	129.46	.000***	.83	164.88	.000***	.86
Communication	59.88	.000*	.70	101.43	.000*	.80
Socialization	170.35	.000*	.87	182.33	.000*	.88
Interpersonal relationships	70.31	.000*	.73	99.91	.000*	.79
Play and leisure time	22.51	.000*	.51	44.42	.000*	.63
Coping skills	90.84	.000*	.78	91.16	.000*	.78
Maladaptive behavior	40.59	.000*	.61	51.97	.000*	.67
CBCL total	9.98	.004**	.28	12.17	.002**	.32
Withdrawal	1.31	.262	.05	2.01	.168	.07
Somatization	8.10	.009	.24	5.82	.023	.18
Anxiety/depression	3.18	.086^†^	.11	3.87	.060†	.13
Social problems	3.13	.089^†^	.11	4.27	.049	.14
Thought problems	6.70	.016	.21	2.75	.109	.01
Inattention	5.08	.033	.16	12.61	.001*	.33
Delinquent behavior	4.95	.035	.16	4.35	.047	.14
Aggressive behavior	4.05	.055^†^	.14	2.68	.113	.09
Internalizing problems	4.03	.055^†^	.13	6.85	.015	.21
Externalizing problems	7.76	.010	.23	5.83	.023	.18

The Wilcoxon signed-rank test was used to assess changes in the QPQ-A/P total get-togethers (the results are presented in the main text)

For the subscales, statistical significance was tested based on adjusted p-values calculated using the Bonferroni–Holm correction. Therefore, asterisks might not be shown, even with a p-value < .05

*p < .05; **p < .01; ***p < .001; ^†^*p *< .10

On the basis of the results regarding the three measures (TASSK, SRS-2 total, and VABS-2 composite) except for SCQ current in Table 5, we confirmed that the differences between two time (pre- and post-test, and pre- and follow-up test) corresponded to a large-sized effect (η_p_^2^ = .28–.86). We recruited 28 participants and used a two-tailed test (α = .05) to compare changes in the means about all measures. The power of these tests for detecting a large effect, under assumed conditions (normality, independence of observations, homogeneity of variance), was over .95. On the other hand, regarding the change in the SCQ current score from pre- to post-test, although the effect of PEERS program was slightly small (middle-sized effect: η_p_^2^ = .09), it achieved sufficient power levels of .89.

Based on the result that there were significant group differences in the baseline scores for one measure (TASSK), the treatment effect was examined using a mixed-design analysis of covariance (ANCOVA) with the above scores taken as covariates. Before that, we first tested whether the assumptions underlying the ANCOVAs had been met. There were two assumptions: (1) the independence of the covariate and treatment effects, and (2) the homogeneity of regression slopes. According to the results of testing these assumptions on all outcome measures, four measures (VABS-2 composite, VABS-2 socialization, VABS-2 play and leisure time, and VABS-2 interpersonal relationships) met both assumptions. Thus, for these measures, two-way mixed ANCOVAs were performed with group (TG/DCG) as the between-participant factor and score (pre-test/post-test, pre-test/follow-up) as the within-participant factor. The results revealed significant effects on VABS-2 composite between pre- and post-test (*F*(1,24) = 8.80, *p *= .007) and between pre-test and follow-up (*F*(1,24) = 12.13, *p *= .002), on VABS-2 socialization between pre- and post-test *(F(*1,24) = 6.19, *p *= .020) and between pre-test and follow-up (*F*(1,24) = 7.67, *p *= .010), on VABS-2 interpersonal relationships between pre- and post-test (*F*(1,24) = 12.17, *p *= .002) and between pre-test and follow-up (*F*(1,24) = 15.43, *p *= .001), and on VABS-2 play and leisure time between pre-test and follow-up (*F*(1,24) = 8.65, *p *= .007).

In the next analysis, McNemar’s test was used to evaluate how many participants (TG and DCG combined) above the cutoff points at baseline had changed to below the cutoff points after treatment (Table 6). The number of participants scoring below or at/above the cutoff points at pre-test, post-test, and follow-up were then calculated. Regarding the VABS-2 composite consisted of three subscales, we used adjusted p-values based on the Holm correction. Five measures were examined: CBCL, DSRS-C, SRS-2, SCQ, and VABS-2 (composite/communication/socialization/maladaptive behavior). Differences in the proportion of those meeting and not meeting the cutoff points were compared between pre- and post-treatment and between pre-treatment and follow-up. The findings revealed that the VABS-2 showed significant improvement between pre- and post-treatment and between pre-treatment and follow-up. CBCL and SRS-2 also showed significant differences between pre-treatment and follow-up.

**Table 6:** McNemar’s test for the number of participants scoring below and above the cutoff point for variables in the TG and DCG combined (%)

Variable	Pre-		Post-		Follow-up		Pre–Post		Pre–follow-up	
	Below cutoff	Above cutoff	Below cutoff	Above cutoff	Below cutoff	Above cutoff	χ^2^ (1)	*p*	χ^2^ (1)	*p*
CBCL	9 (32.1)	19 (67.9)	13 (46.4)	15 (53.6)	15 (53.6)	13 (46.4)	9.614	0.219	11.495	0.031*
SRS-2	5 (17.9)	23 (82.1)	5 (17.9)	23 (82.1)	11 (39.3)	17 (60.7)	16.025	1.000	9.407	0.031*
SCQ current	25 (89.3)	3 (10.7)	24 (85.7)	4 (14.3)	25 (89.3)	3 (10.7)	20.160	1.000	28.000	1.000
DSRS-C	17 (60.7)	11 (39.3)	18 (64.3)	10 (35.7)	22 (78.6)	6 (21.4)	6.152	1.000	6.212	0.125
VABS-2 composite	1 (3.6)	27 (96.4)	12 (42.9)	16 (57.1)	17 (60.7)	11 (39.3)	1.383	0.001***	0.671	0.000***
Communication	5 (17.9)	23 (82.1)	14 (50.0)	14 (50.0)	20 (71.4)	8 (28.6)	6.087	0.004*	2.435	0.000*
Socialization	3 (10.7)	25 (89.3)	15 (53.6)	13 (46.4)	22 (78.6)	6 (21.4)	2.912	0.000*	0.916	0.000*
Maladaptive behavior	11 (39.3)	17 (60.7)	25 (89.3)	3 (10.7)	25 (89.3)	3 (10.7)	2.174	0.000*	2.174	0.000*

SRS-2: the score of 60 (middle and high risk level) was used as a cutoff

VABS-2: the classification of severity for disabilities was used as a cutoff since there were no cutoff scores

VABS-2 communication ≤ 70 (low adjustment level)

VABS-2 socialization ≤ 70 (low adjustment level)

VABS-2 maladjustment ≥21 (high maladjustment level)

Statistical significance was determined using adjusted p-values based on the Holm correction for the VABS-2 composite subscales

*p < .05; **p < .01; ***p < .001

Finally, the Wilcoxon signed-rank test was used to assess changes in the number of get-togethers as assessed by the QPQ. Since the assumption of normal distribution was violated, using an independent t-test was considered inappropriate. The Japanese version of the QPQ includes the number of get-togethers that happened both inside and outside of the home. A comparison between pre- and post-test (TG and DCG combined) revealed a significant increase in the number of get-togethers occurring both inside and outside of the home, as reported by both adolescents (*Z *= −1.999, *p *= .046) and parents (*Z *= −2.147, *p *= .032), while no significant change was observed from pre-test to follow-up: adolescents (*Z *= −1.474, *p *= .141) and parents (*Z *= −1.312, *p *= .190).

## Discussion

The goal of the present study was to examine the treatment effectiveness of PEERS in Japan. The overall findings indicated that with minor cultural changes, PEERS is effective in improving social skills related to making and keeping friends for adolescents with ASD in Japan.

The first hypothesis, that adolescents in the TG would show significant improvements following treatment compared with adolescents waiting for treatment in the DCG, was supported in the areas of knowledge of social skills (TASSK), adaptive functioning (VABS-2 composite), socialization (VABS-2), play and leisure time (VABS-2), and maladaptive behavior (VABS-2). The improvement in social skills as indicated on the TASSK after the intervention was comparable to that in most previous studies (Gantman et al. 2012; Laugeson et al. 2009, 2012; Schohl et al. 2013; Yoo et al. 2014; Shum et al. 2018). In those studies, autistic traits (SRS-2) improved significantly after the treatment, whereas in the present study, improvement was seen at the follow-up assessment. Interestingly, pre- and post-test comparisons of communication and interpersonal relationships (VABS-2) showed meaningful improvement in both the TG and DCG, which although unexpected, might be related to maturation and the adolescents’ natural development during this period of time.

The second hypothesis, that adolescents in both groups would show improved social skills after the treatment, was verified in the following areas: socialization (VABS-2), communication (VABS-2), knowledge of social skills (TASSK), autistic traits (SRS-2), and behavioral and emotional problems (CBCL total and VABS-2). On most of the CBCL subscales, no significant changes in scores from pre-test to post-test or from pre-test to follow-up test were observed (see Table 5). We gave first priority to maintain suitable alpha level. Therefore, we had to set stringent alpha level for hypothesis testing on the CBCL subscales. As a result, the power of the test to detect a true alternative hypothesis would rather be reduced. The specific areas of improvement are discussed further below.

Regarding the area of socialization, both groups showed significant improvement on the VABS-2 following treatment, not only in overall adaptive functioning (composite score), but also in play and leisure time, coping skills, and interpersonal relationships. These improvements make sense given the emphasis on play skills and good sportsmanship indicative of the PEERS intervention. For example, in Sessions 8 and 9, didactic lessons related to having successful get-togethers and being a good sport are presented. For the remaining sessions, therapists and coaches repeatedly review the elements for having successful get-togethers, along with the meaning of good sportsmanship during the homework review and behavioral rehearsal activities. Thus, nearly half of the intervention emphasizes and encourages adolescents to use rules of play and leisure skills during in-group and out-of-group practice. Through these practice attempts, adolescents apply each skill to real-world situations, thereby gaining confidence and competence in using those skills. Since PEERS is a group-based training program, as participants begin to acquire new skills, they naturally and gradually begin to behave more cooperatively toward each other during behavior rehearsal activities. Furthermore, weekly homework assignments require them to collaborate with other group members, such as during in-group telephone conversations, as well as with peers not affiliated with the program. Previous studies suggest that these elaborate and structured behavioral rehearsal exercises are what render PEERS effective (Gantman et al. 2012; Laugeson et al. 2009, 2012; Yoo et al. 2014). Regarding the improvement observed in socialization, the number of get-togethers occurring both inside and outside of the home increased significantly after the treatment, as reported by both adolescents and parents (QPQ); however, the QPQ result might have been affected by the design of analysis combining data from two groups.

As for the area of communication, the VABS-2 and SRS-2 revealed improvement from pre- to post-treatment. From the very first session of the intervention, adolescents were taught how to engage in conversation with others, such as trading information, maintaining two-way conversations, and even entering and exiting conversations. By helping to decode the formal rules and steps of conversational skills used by socially successful adolescents, participants became aware of the goal of conversations (i.e., to find common interests), allowing them to follow the topic of conversations cooperatively, interactively, and enjoyably. Consistent with this finding, previous studies also show significant improvements in the area of social communication following PEERS (Gantman et al. 2012; Laugeson et al. 2012, 2015).

Improvements in the areas of socialization and communication are especially meaningful for individuals with ASD. According to a survey standardizing the Japanese version of the VABS-2 (2014), the average scores for Japanese youths with ASD indicate significant difficulties in the areas of socialization and communication, regardless of IQ. Therefore, the present finding that PEERS in Japan was shown to be effective in improving communication and socialization is quite promising, since these skills are particular areas of weakness for adolescents with ASD; however, the result of VABS-2 might have been affected by some biases. For example, parents were active participants in this program, and the interviewer was a member of the research team.

Knowledge of social skills involves the understanding of basic rules and steps of social etiquette, and is an essential ingredient in adolescents’ taking their first steps toward improving social functioning (Gardner et al. 2015). In the present study, knowledge of social skills increased significantly from pre- to post-assessment on the TASSK. One simple explanation for this finding involves the use of Socratic questioning in the adolescent sessions. Adolescents were not lectured to by an instructor, but instead, were shown role-play demonstrations or asked questions in such a way that they must generate rules and steps for social behaviors. As a result, the adolescents not only feel like social skills experts, but also are far more likely to believe what they are taught and remember what they learn, thereby enhancing their knowledge of social skills. Moreover, using buzzwords and phrases to teach social skills is arguably an effective technique for learning complex social rules. For example, during the PEERS sessions, each rule or strategy was taught through the presentation of buzzwords, such as “trade information” to “find common interests”, while avoiding being “a conversation hog” or “an interviewer”, etc. Gradually, these buzzwords became common language used by parents, adolescents, and the treatment team to talk about sophisticated social behavior succinctly and yet with depth. Since parents also learned the same buzzwords and phrases, they were able to provide focused social coaching during teachable moments in the real world, enhancing memorization and generalization of skills. The use of buzzwords taught using a Socratic method likely contributed to the adolescents in the present study using the skills consciously, thereby increasing their knowledge of social skills over the course of treatment. This finding is in accordance with previous studies in both North America and Asia (Laugeson et al. 2009, 2012; Mandelberg et al. 2014; Schohl et al. 2013; Yoo et al. 2014).

Another important outcome of the present study was a decrease in autistic traits related to social responsiveness following treatment, especially in the areas of social awareness, social cognition, social communication, and autistic mannerisms, as measured by the SRS-2. Autistic mannerisms relate to behaviors such as perseverating on restricted interests, while disregarding the other person’s interests in conversation. The decrease in autistic symptoms might help adolescents in PEERS attain better social functioning in daily life, in addition to successful social interactions (Schohl et al. 2013). While the PEERS curriculum does not target autistic mannerisms per se, the emphasis on having reciprocal two-way conversations and finding common interests is a cornerstone of the program, and may be attributable to the decrease in autistic traits observed in the present as well as previous studies (Gantman et al. 2012; Laugeson et al. 2012, 2014, 2015; Yoo et al. 2014).

Although PEERS does not specifically target behavioral and emotional problems, some positive effects were shown following treatment, as measured by parent reports on the CBCL (from pre- to post-test and to follow-up test) and DSRS-C (from pre- to follow-up test). With the risk of developing depression and/or anxiety disorders heightened within this population (Sadolescentsel and Heeman 2017), improvement in behavioral and emotional health is very meaningful. In fact, effective treatment to promote anxiety reduction and social skills development for adolescents with ASD is widely thought to be an important component to improve social functioning (White et al. 2010). Even though anxiety reduction is not the targeted goal of PEERS, improvement in socialization and communication would naturally be helpful for increasing confidence in relating with others, thereby reducing social anxiety. PEERS has been shown to be effective in decreasing social anxiety (Schohl et al. 2013), perhaps in part because of its use of CBT methods, which have been reported to be an effective approach for treating co-occurring anxiety in adolescents with ASD (White et al. 2009b). Although changes in emotional and behavioral problems following PEERS have been studied to a lesser extent in North America, adolescents in the present study and in the Korean study (Yoo et al. 2014) demonstrated improvements in these areas.

The efficacy of PEERS was demonstrated under substantial test power. Nevertheless, a significant change of SCQ score was not shown in pre- and post-test, as contrasted to pre- and follow up-test. Therefore, it suggests that in a relatively short period of time PEERS program has a difficulty to alleviate the feature of ASD on social communication. In other words, the development of social communication through PEERS program gradually appears. As an exception, the power of test on the DSRS-C was insufficient (the power is less than .80). The power analysis shows that a sample of at least n = 40 will be needed to reach power of .80. Further experiment, which achieved a necessary condition, would be required to decide whether PEERS program has an effect on the DSRS-C score or not.

The third hypothesis that positive treatment effects would continue after the intervention, was also supported by the results of the present study in all of the above areas. In addition to the maintenance of treatment gains, several new improvements were observed at follow-up in the areas of decreased autistic traits (SCQ) and depressive symptoms (DSRS-C). These results are likely the result of parental involvement in treatment through social coaching in the home and community, which is thought to enhance the generalization and durability of social skills gains (Laugeson et al. 2014, 2015; Miller et al. 2014). By including parents in the intervention, the notion is that treatment never ends, even in the context of time-limited therapy (Laugeson et al. 2014).

The fourth hypothesis, that the number of adolescents with clinically severe mental health conditions would decrease following treatment, was supported in three areas: behavioral and emotional problems (CBCL), autism symptoms/social responsiveness (SRS-2), and adaptive functioning in relation to communication and socialization (VABS-2). Within these domains, the number of adolescents who manifested severe symptoms at baseline significantly decreased after treatment, suggesting that the treatment lightened the severity of these problems as a secondary outcome. This finding is of particular salience given the fact that children and adults with ASD in Japan whose SRS-2 scores reveal moderate symptoms/risk (T-score 60–75) were 13 times more likely to have psychiatric problems and six times more likely to experience emotional problems compared with the low symptom/risk group (T-score < 60) (Kamio et al. 2013b). In the present study, the number of adolescents with moderate or high symptom/risk (T-score: ≥ 60) prior to treatment decreased significantly following treatment, suggesting that the intervention contributed to the lessening of secondary difficulties. As the data from two groups were combined for this hypothesis, the result needs be carefully examined.

As previously mentioned, despite the cultural and linguistic differences, the overall findings from the present study were very similar to those in previous PEERS studies. In addition to the minor cultural modifications made prior to the intervention, the research team also acquired feedback from parents and adolescents while implementing the treatment. The conclusion of this ongoing needs assessment revealed that the social skills presented in the curriculum were acceptable to Japanese adolescents and parents. The face validity of the cultural adaptations is encouraging because it suggests that this program should be acceptable to Japanese youths. However, one cultural difference related to adolescent lifestyle might be considered in future research. The results of a comparative study examining how children and adolescents spend free time across the world (Larson and Verma 1999) suggest that East Asian adolescents spend more time on school studies and less time on leisure activities than their North American counterparts. This finding is in accordance with feedback from the present study, which resulted in the addition of a supplementary item in the questionnaire assessing the frequency of get-togethers outside of the home to include social interactions in the community. Future research might also take cultural aspects of adolescent lifestyles into consideration when assessing treatment effects.

According to the feedback from parents and adolescents just after the treatment finished, adolescents felt more confident in interacting with others, and the number of get-togethers increased. In addition, adolescents seemed to have become more aware of themselves in a difficult situation and to have started understanding how they could approach those scenarios. The skills that both parents and adolescents felt were especially useful were: “Tease the tease”, “Handling disagreement”, “How to handle bullying and bad reputations”, “Trading information”, “Entering and exiting a conversation”, and “Two-way conversations”. Parents stated that, as a social coach, helping adolescents tackle homework was not an easy task because some homework was closely related to their difficulties; however, this is the reason they had a feeling of accomplishment and actually learned skills after they had finished.

Although the results of the present study are encouraging, they are limited in several ways. The first limitation relates to the study design. Unlike the majority of studies examining the efficacy of PEERS in North America, this study was not a randomized controlled trial. Although the research team had intended to randomize the sample, due to the scheduling constraints of the students, participation in one group or the other was determined by convenience. For example, some of the students could not be assigned to the DCG because their schools had a Saturday class, or they were in the ninth grade, so they had to prepare for the entrance exam to high school. Therefore, we could not eliminate the possibility of an unintended allocation bias. Future research in Japan with larger samples might include randomization of assignment to groups to avoid such bias. The second limitation relates to the lack of blinded assessments, in that the outcome measures reported by the parents might have been biased, as parents were active participants in the program. In addition, the interviewer was one of the study team members. With more funding for this research, we might have been able to reduce the possibility of bias by hiring an interviewer for the semi-structured interviews. This limitation could have affected the results, for example, VABS-2 composite scores, which increased broadly. Therefore, care must be taken when considering the results. Although there was a seemingly unusual increase in the composite score, the changes in the subscales were very similar to those reported in a previous Korean study (Yoo et al. 2014). Similar to previous studies in North America, future research in Japan might need to include independent ratings of social functioning from blinded teachers who are unaware of a student’s participation in social skills programs. Additionally, behavioral observation measures, such as the Contextual Assessment of Social Skills (Ratto et al. 2011), might provide more objective findings in future research. The third limitation is related to the profiles of the two groups before receiving the treatment. There was originally a group difference in social skills knowledge (TASSK) assessed at baseline. Additionally, in the DCG, there was a slight score increase for communication and interpersonal relationships (VABS-2) from Time 1 to Time 2, even without treatment. This means that we could not totally exclude the possible influence of their original property and their spontaneous change. The fourth limitation is in relation to the research design of QPQ for hypotheses 3 and 4, and McNemar’s test regarding the differences in proportions of adolescents with and without severe mental conditions. We combined the two groups to test these hypotheses with no experimental control. Therefore, the results regarding the positive score changes in skills or symptoms and the decreased number of adolescents with severe conditions require careful interpretation. The fifth limitation relates to the recruitment criteria for this study. For example, the inclusion criteria required a student to exhibit no disruptive behavior, be motivated to participate, and have adequate cognitive and language ability to understand the instructions, etc. Thus, the present results regarding the effect of PEERS in Japan might not be able generalizable to adolescents who could not meet these criteria. The sixth limitation relates to the small sample size in the present study. If a larger sample could be collected, the treatment effects may strengthen the validity. The seventh limitation relates to the checklist for video fidelity. Unfortunately, the qualitative data was not collected on treatment fidelity. Instead, all staff members were trained on the protocol, had reviewed the manual thoroughly before each session, and received supervision by two supervisors every week. Although we made the greatest effort to follow the manual as presented, having quantitative data would have strengthened the validity of this study. A final limitation of the present research relates to the characterization of the sample. Because of the financial constraints of the study and the shortage of qualified assessment staff, only 10 of the 28 participants were administered the ADOS-Second Edition (ADOS-2; Lord et al. 2012), a standardized diagnostic measure for autism. Although the lack of diagnostic assessment using the gold-standard autism diagnostic tool is a limitation of the present study, it is important to note that all participants had a previous diagnosis of ASD from a reliable medical or mental health professional, confirmed by elevated scores reaching clinical significance on the SRS-2, which has been found to be sufficient to establish a diagnosis (Laugeson et al. 2009, 2012; Gantman et al. 2012; Laugeson et al. 2015).

In conclusion, despite the limitations of the present study, the findings suggest that PEERS is effective for improving social functioning in Japanese adolescents with ASD. The results are in accordance with those from previous studies examining the efficacy of PEERS in Asia (Yoo et al. 2014; Shum et al. 2018) and North America (Laugeson et al. 2009, 2012; Schohl et al. 2013; Laugeson et al. 2014). Similar positive results in combination with minimal cultural adaptations suggest that the basic strategies needed for making and keeping friends in the United States basically apply to Japanese youths. In future research, the cultural aspects of friendship in Japan should be analyzed in greater detail in association with the ASD population. In addition, 1–5 years of follow-up after an intervention would be beneficial to examine the long-term effectiveness of the program. Further cross-cultural validation trials regarding the efficacy and effectiveness of PEERS using school-based programs (Laugeson 2014) and young adult programs (Laugeson 2017) are needed, and could be expected to be valuable toward meeting the needs of the larger ASD population in Japan.
